# Ageing of juvenile coral grouper (*Plectropomus maculatus*) reveals year-round spawning and recruitment: implications for seasonal closures

**DOI:** 10.1098/rspb.2023.0584

**Published:** 2023-06-28

**Authors:** H. B. Harrison, L. Drane, M. L. Berumen, B. J. Cresswell, R. D. Evans, G. F. Galbraith, M. Srinivasan, B. M. Taylor, D. H. Williamson, G. P. Jones

**Affiliations:** ^1^ School of Biological Sciences, University of Bristol, 24 Tyndall Avenue, Bristol BS8 1TQ, UK; ^2^ Marine Biology and Aquaculture, College of Science & Engineering, James Cook University, Townsville 4811, Queensland, Australia; ^3^ Centre for Tropical Water and Aquatic Ecosystem Research (TropWATER), James Cook University, Townsville 4811, Queensland, Australia; ^4^ Red Sea Research Center, Division of Biological and Environmental Science and Engineering, King Abdullah University of Science and Technology, Thuwal 23955, Saudi Arabia; ^5^ Commonwealth Scientific and Industrial Research Organisation, Townsville 4811, Queensland, Australia; ^6^ Department of Biodiversity Conservation and Attractions, 17 Dick Perry Ave, Kensington 6151, Australia; ^7^ Oceans Institute, The University of Western Australia, Crawley, Western Australia 6009, Australia; ^8^ University of Guam Marine Laboratory and UOG Sea Grant, 303 University Drive, UOG Station, Mangilao, Guam 96923, USA; ^9^ Great Barrier Reef Marine Park Authority, Townsville 4810, Queensland, Australia

**Keywords:** seasonal fishing closures, reproductive ecology, coral reef fisheries, age-based demography

## Abstract

Temporal patterns in spawning and juvenile recruitment can have major effects on population size and the demographic structure of coral reef fishes. For harvested species, these patterns are crucial in determining stock size and optimizing management strategies such as seasonal closures. For the commercially important coral grouper (*Plectropomus* spp.) on the Great Barrier Reef, histological studies indicate peak spawning around the summer new moons. Here we examine the timing of spawning activity for *P. maculatus* in the southern Great Barrier Reef by deriving age in days for 761 juvenile fish collected between 2007 and 2022, and back-calculating settlement and spawning dates. Age-length relationships were used to estimate spawning and settlement times for a further 1002 juveniles collected over this period. Unexpectedly, our findings indicate year-round spawning activity generates distinct recruitment cohorts that span several weeks to months. Peak spawning varied between years with no clear association with environmental cues, and little to no alignment with existing seasonal fisheries closures around the new moon. Given the variability and uncertainty in peak spawning times, this fishery may benefit from additional and longer seasonal closures, or alternative fisheries management strategies, to maximize the recruitment contribution from periods of greatest reproductive success.

## 1. Introduction

Marine fish populations are characterised by juvenile recruitment patterns that are highly variable in space and time. For coral reef fishes, considerable attention has been given to understanding spatial variation in recruitment patterns and its impact on the distribution and abundance of a species [1–4]. Recruitment patterns can often vary substantially among locations leading to recruitment hotspots or places that consistently experience reliable levels of recruitment and high population densities [5,6]. Such spatial heterogeneity in the recruitment of reef fishes is often driven by strong habitat preferences at the time of settlement to the reef [7,8]. For exploited species, spatial management strategies such as no-take marine reserves that target recruitment hotspots may be particularly effective in enhancing fish stocks [9].

In comparison, there have been fewer studies on temporal variation in the recruitment of coral reef fishes and its management implications. Recruitment is extremely variable among and within years [1,10,11]. Such variation can lead to fluctuations in adult population sizes and make fishery stock-recruitment relationships extremely unpredictable [12,13]. There are likely to be multiple causes of temporal variation in recruitment, including the timing of spawning activity [14], variations in somatic growth and survival of larval stages [7], and temporal variation in larval supply from different source populations [15], all of which can have a large impact on the number of individuals recruiting to the adult population of a given reef. However, in general the causes of temporal variation in recruitment are poorly understood.

As most coral reef fishes have a restricted pelagic larval duration (PLD) [16–18], temporal patterns in spawning are likely to be a major driver of temporal patterns in recruitment [11,19,20]. On some low latitude coral reefs, fish spawn and recruit throughout the year [21]. However, most coral reef fishes exhibit distinct spawning seasons, which vary by regions and by latitudes, with shorter breeding seasons at high latitudes [22–24]. Distinct spawning seasons are thought to occur during months where environmental conditions maximize the survival and performance of their offspring [25]. The timing and duration of spawning seasonality in coral reef fishes have been linked to temperature cycles [21,26]. Other studies have shown weak associations between rainfall and reproductivity, some negative [21,26] and others positive [27]. The effects of temperature and rainfall may be species and location specific. A large proportion of coral reef fishes also exhibit distinct lunar spawning cycles within spawning seasons, often during new or full moon phases [23,25,28]. Lunar synchrony may occur at times that reduce predation on larvae or enhance reproductive success, which may enhance recruitment within spawning seasons [14]. Spawning during different lunar phases can have a major influence on pre- and post-settlement growth and survivorship [28–33]. However, the timing of spawning in relation to annual and lunar cycles is not known for the vast majority of reef fish species.

The timing of spawning is particularly important in relation to temporal fisheries management strategies such as seasonal closures. It is surmised that recruitment to the fishery can be enhanced by halting fishing during peak times of the spawning season and lunar cycle [34,35]. On this basis, the Queensland Line Fishery (Reef) is subject to short seasonal closures during summer new moons based on the presumption of seasonal and lunar spawning of the common coral grouper *Plectropomus leopardus* [36,37]. The fishery closure applies to both commercial and recreational sectors and covers a range of reef fish species including common coral grouper (*P. leopardus*), bar-cheek coral grouper (*P. maculatus*), and other Serranidae, Labridae, Lutjanidae and Lethrinidae species [37–40]. However, it is not known whether all these species, across all regions of the Great Barrier Reef (GBR), exhibit the same seasonal and lunar spawning patterns, and so the effectiveness of the seasonal closure for the whole fishery has not been assessed.

Direct observations of spawning activity to define spawning seasons and lunar cycles are not always possible and the histological assessment of seasonal gonad development provides only limited indication of spawning activity and the frequency of spawning events. Even when spawning events are directly observed, knowing where larvae go and successfully settle, and recruit is challenging. Individuals that have already recruited into a population could therefore be considered more demographically relevant to quantify reproductive activity. Direct examination of these individuals and the temporal patterns of their cohorts can be used to hindcast the timing of successful spawning activity, allowing inference of environmental cues for spawning and the best times to apply seasonal closures to enhance recruitment. Here we use age estimates of a large sample of juvenile coral grouper (*P. maculatus*) collected at regular intervals from fringing reefs of a small island archipelago in the southern GBR to back-calculate the date of hatching of juvenile fish (less than 250 mm) that have successfully settled and recruited to local reefs. We then inferred peaks in spawning activity across multiple years that we could relate to environmental conditions and investigate the effectiveness of seasonal closures for the coral grouper fishery on the GBR.

## 2. Methods

### (a) Study site and sample collection

The Keppel Islands are an inshore island archipelago of the southern Great Barrier Reef Marine Park, popular among recreational fishers. Prior to 2009, all fishing activity was closed for three nine-day periods during the new moon phases of late spring and early summer (October to December). Changes in legislation reduced the number and duration of fisheries closures to two five-day periods during the same period [41].

Juvenile *P. maculatus* were sampled from reefs throughout the Keppel Islands (23.18°S, 150.95°E) during three multi-year collection periods spanning 15 years (2007–2022), with 2–3 sampling trips within each period (electronic supplementary material, table S1). Individuals up to 250 mm total length were collected on SCUBA using either spearguns or hand spears, with some smaller fish up to 50 mm collected using clove oil and hand nets. In total, 1763 juveniles were collected and the fork length (FL) and total length (TL) of each fish was measured to the nearest millimeter and weighed to the nearest 0.1 gram.

### (b) Otolith preparation and age determination

The sagittal otoliths were extracted from a subset of individuals from each collection period (electronic supplementary material, table S1) to estimate age and age-length relationships of juvenile *P. maculatus*. The preparation of otoliths followed the methods described in [42] and is consistent with previous studies of early life-history growth in *P. maculatus* from the southern GBR [15,43,44]. Briefly, one otolith from each juvenile fish was affixed to a glass microscope slide using thermoplastic glue (Crystalbond 509), with the primordium (nucleus) on the inside edge of the slide and sulcus ridge perpendicular to the slide edge to obtain a transverse section of the sagittal otolith. Each otolith was ground to the edge of the slide using a GEMMASTA lapping wheel with a 1200 grit diamond sanding wheel. The otoliths were then removed and affixed to a clean labelled slide, with the ground surface down, and polished using the same grit to a thin (≃150 µm) transverse section that intercepts the nucleus. Successive polishing was then carried out with 9, 3 and 0.3 µ lapping film until daily growth increments were of optimal clarity. Sectioned otoliths were then coated in immersion oil and photographed under 200× and 400× magnification.

### (c) Calculating the date of hatching of aged juveniles

The post-settlement age of each aged juvenile was estimated through three independent counts of the daily growth increments, from the settlement mark to the outer edge along the ventral surface, following the longest plane. The final post-settlement age was taken from the mean of the three counts, when each of the three counts were within 10% difference of the median. Samples with counts greater than 10% of the median were excluded from the analysis. To reduce the possibility of potential observer effects in counts, a sub-sample of 50 otoliths across sampling periods were cross-validated and verified by the same observer (BMT) and found to be consistent between observers.

In total, 761 juvenile *P. maculatus* were aged, ranging from 23 mm to 248 mm in total length with a mean length of 116.6 mm (electronic supplementary material, table S1, electronic supplementary material, figure S1*a*). The pelagic larval durations (PLDs) were estimated for 70 individuals by counting daily age increments from the primordium to the settlement mark of the otolith (electronic supplementary material, figure S1b), with a mean PLD of 27.9 days ± 1.6 s.d. We calculated the date of hatching of aged juveniles by subtracting their measured post-settlement age and mean PLD from the date of collection. Approximately 43% of juvenile fish were aged across all sampling periods (electronic supplementary material, figure S2) to account for variation in early growth between years.

### (d) Estimating the date of hatching of non-aged juveniles

The age–length relationship of 761 aged juvenile fish was used to determine the settlement date of a further 1002 non-aged juveniles up to 250 mm in total length. First, six discrete cohorts were visually identified from the distribution of spawning times of aged juveniles. (electronic supplementary material, figure S3). Preliminary analyses identified small but significant variation in the age-length relationships between cohorts. Therefore, a generalized linear mixed effects model with a third order polynomial structure was used to model post-settlement age against total length with a Gaussian error structure controlling for pre-defined cohorts (random effect) (electronic supplementary material, figure S4 and table S2). We included a dispersion factor for *total length* and *cohort* to control for heteroscedasticity in the residuals plots due to decreasing ageing precision with total length and minimize its effect on the model predictions. The data fit the assumptions of the model with homogeneity of variance and no dispersion or outliers.

Secondly, non-aged juveniles were assigned to the six cohorts defined above based on the time at which they were collected. These were visually checked for accuracy. Then, using the age-length relationship estimated above, we predicted the age of 1002 non-aged juvenile fish based on their total length, accounting for variation in early somatic growth between cohorts. The age of juvenile fish that were collected outside of the pre-defined cohorts was estimated from the marginalized mean of all cohorts (electronic supplementary material, figure S4).

Finally, the time of settlement was determined by subtracting the age of each fish from the time of collection. We then subtracted the mean PLD from the time of settlement to estimate the time of hatching of each juvenile fish that successfully recruited to the island group. Calculations did not account for the embryonic development of fertilized eggs, which is typically 11 to 20 h post-fertilization in *P. leopardus* hatcheries [45].

All GLMMs were performed using the *glmmTMB* package in R [46]. Model residuals were inspected in the package *DHARMa* [47] and checked for homogeneity of variance, dispersion, and outliers. Model predictions were performed and visualized with *emmeans* [48] and *tidyverse* [49], and summarized with *broom.mixed* [50]. All models and graphics were conducted within the R statistical and graphical environment [51]. Where relevant, confidence intervals were based on a 95% significance level.

### (e) Temporal spawning patterns

The time of hatching of juvenile fish was used to infer spawning activity of *P. maculatus* at the Keppel Islands from 2007–2022. We used generalized additive models (GAMs) within the package *mgcv* [52] to identify peak spawning times for five austral years (July–June). Periods spanning 2006–2007, 2010–2011 and 2019–2020 were not considered due to insufficient data (4 individuals removed). In addition, 79 individuals from 2012–2013 were removed from further analyses due to a combination of low sample numbers and lack of ageing data to predict age-length relationships for this period. The number of fish spawned in 5-day windows were used as the response variable that assumed a Tweedie error distribution with a logarithmic link-function to account for over-dispersion caused by periods with no spawning activity. The GAMs were tested for each austral year based on the following formula:

$$y=\beta_{0}\;+\;(\mathrm{time})+\varepsilon ,\;\;\varepsilon ∼Tw_{p}(\mu ,\sigma^{2}),$$

where *β*_0_ is the average number of individuals spawned in a 5-day period (intercept) and (*time*) is the smoothing function for the annual trend in spawning. DHARMa residuals were checked for homogeneity of variance, dispersion, and outliers. Additional tests for zero inflation, overdispersion and over-smoothing were performed to satisfy model fit. Model selection was informed from the Akaike information criterion (AICc) with the lowest score [53]. Spawning peaks were identified from the first derivative of the fitted GAM functions and plotted with partial residuals. The model predictions were used to identify peaks in spawning activity, the duration between peaks, and to quantify the likely contribution of spawning closures between October and December of each year.

### (f) Environmental drivers of spawning activity

We also explored the environmental conditions associated with spawning activity using GAMs that included lunar illumination, Sea Surface Temperature (SST), local rainfall and flood gauge data from the Fitzroy River as covariates. The number of individuals spawned were summed over 5-day periods to minimize the influence of zero-values in the data. Lunar illumination values were averaged over the same 5-day period using the *lunar* package [54] with a +10-hour shift to account for Australian Eastern Standard Time. SST values were generated by the Giovanni online data system developed and maintained by the NASA GES DISC [55], which generates an 8-day average of night-time SST collected by the MODIS-Aqua satellite sensing system. Rainfall and flood data were generated from the Australian Government Bureau of Meteorology data portal.

The number of individuals spawned in a 5-day period were used as the response variable for GAMs that assumed a Tweedie error distribution with a logarithmic link-function to account for overdispersion caused by periods with no spawning activity. We explored models with a spline fitted to each covariate and the possible interaction between lunar phase and SST. Model selection was informed from the AICc and model fit. The best model included an interaction between SST and month, marginalized over years and was based on the following formula:

$$y=\beta_{0}\;+\;(SST|\mathrm{Month})+\gamma_{\mathrm{year}}+\varepsilon ,\;\varepsilon ∼Tw_{p}(\mu ,\sigma^{2}),$$

where *β*_0_ is the average number of individuals spawned in a 5-day period (intercept), (*SST*|Month) indicates the additive smoothing functions of the interaction between SST and month, and *γ*_year_ indicates the random smoothing term of year. Rainfall and lunar illumination were not important explanatory variables and were excluded from the model. Flood height had a significant effect though showed high concurvity with SST and month (0.77). DHARMa residuals were checked for homogeneity of variance, dispersion and outliers. Additional tests for zero inflation, overdispersion, concurvity and over-smoothing were performed to satisfy model fit.

### (g) Assessing the effectiveness of seasonal fishery closures

Finally, we assessed whether the number and duration of spawning closures from October to December increases the likelihood of capturing a peak in spawning activity. For each year, we calculated the proportion of successful spawns captured during 5-day and 9-day closures around the new moon. A generalized linear mixed effects model was used to model spawning activity against the duration and number of seasonal closures with a Beta error structure marginalized over years (random effect) in the *glmmTMB* package in R. The data fit the assumptions of the model with homogeneity of variance and no dispersion or outliers. We used the modelled relationship to predict and compare the spawning activity captured by each seasonal fishery closure.

## 3. Results

### (a) Temporal patterns in spawning activity

**Figure 1.**
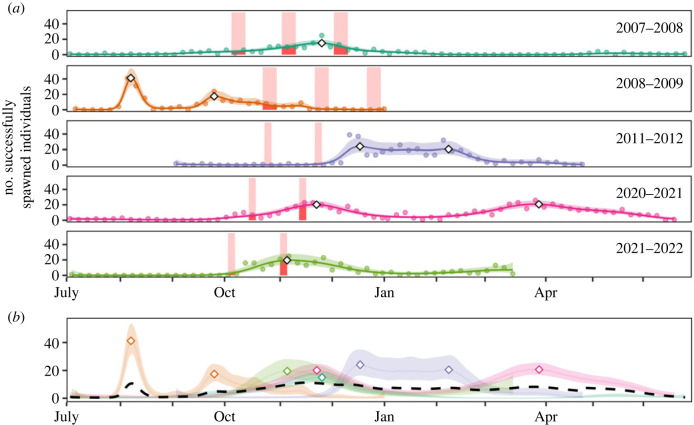
Annual variation in spawning activity of *P. maculatus* at the Keppel Islands inferred from hatching dates of 1763 juveniles. (*a*) Spawning activity that led to the successful recruitment of juveniles *P. maculatus*. In each 12-month period, a GAM model predicted trends in the number of individuals spawned in 5-day windows with 95% confidence intervals. Points represent the observed data and white diamonds identify peaks in spawning activity from the first derivative of the smooth functions. Red bars indicate seasonal fishery closures that coincide with the summer new moons. (*b*) Predicted spawning activity overlayed for each year with the dashed line representing the yearly average number of juveniles spawned in a 5-day window that successfully recruited to reefs at the Keppel Islands.

Year-round spawning activity of *P. maculatus* at the Keppel Islands was derived over multiple years based on the age of 1763 juvenile fish under 250 mm collected from 2007 to 2022. The post-settlement age of 761 juvenile fish was first measured from daily growth increments of sagittal otoliths and used to derive an age–length relationships for juvenile *P. maculatus* that accounted for temporal variation in growth over this period. The post-settlement age of a further 1002 juveniles was then derived from this relationship. Finally, the date individuals hatched was estimated by subtracting the post-settlement age and PLD from the time of collection, thereby providing a unique perspective on the spawning activity of *P. maculatus* at the Keppel Islands. We found spawning activity occurred year-round in distinct cohorts spanning periods of 1–4 months (electronic supplementary material, figure S5). However, the timing of peak spawning activity was not consistent in each year indicating temporal fluctuations in successful spawning (figure 1). This provided the basis to investigate temporal patterns in spawning activity, the environmental conditions that may trigger spawning, and the effectiveness of spawning closures for *P. maculatus* at the Keppel Islands.

GAMs centred on the austral summer were fitted separately to the date of spawning of juvenile *P. maculatus* in each of 5 years. The best fit GAMs yielded robust diagnostics and explained at least 80% of the null deviance depending on the year, indicating good model fits. All years showed evidence of a long-term nonlinear trend in spawning and of similar and significant wiggliness (electronic supplementary material, table S3). The timing of peak spawning varied between years and ranged from August through to March. Each year presented one to two peaks in spawning, with distinct peaks separated by 50 to 127 days (figure 1*a*). Peaks in spawning were of similar strength ranging from 15 to 34 juveniles successfully spawned in a 5-day window (mean: 21.4 95% CI [15.0–30.9]) though ranged in their duration and thus their contribution to local recruitment at the Keppel Islands. Averaged across the 5 years (figure 1*b*), spawning occurred year-round and although there appears to be an increase in spawning activity between October and December, the spawning patterns were too variable to confidently differentiate spawning activity between seasons.

### (b) Environmental drivers of spawning activity

**Figure 2.**
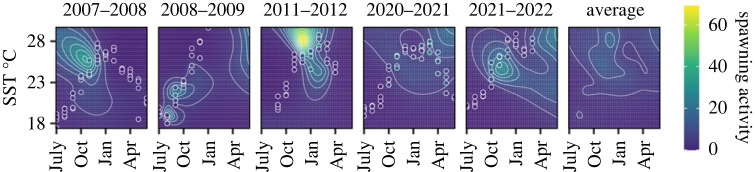
Spawning activity of *P. maculatus* at the Keppel Islands was strongly associated with sea surface temperature (SST) and time of year, though trends were inconsistent between years. Shaded areas and contour lines indicate the predicted spawning activity (in 5-day windows) of *P. maculatus* at the Keppel Islands. Estimates of spawning activity are overlaid in open circles for each year.

We investigated the environmental conditions associated with spawning activity of *P. maculatus* at the Keppel Islands and identified an important interaction between SST and time of year (month). Neither lunar illumination nor rainfall captured variation in spawning activity whereas flood height was highly correlated with SST and time of year and was therefore excluded from the model (electronic supplementary material, figure S6). The best fit GAM included an interaction between SST and month marginalized over years (electronic supplementary material, table S4), which yielded robust diagnostics and explained at least 80% of the null deviance, indicating a good model fit. All years showed evidence of a long-term nonlinear trend in spawning activity associated with SST throughout the year (electronic supplementary material, table S4). When predicting spawning activity throughout the range of SST recorded at the Keppel Islands it is possible to identify clear peaks in spawning activity (figure 2). However, the results were not consistent between years suggesting that additional unknown factors not considered here may influence the timing of spawning activity or survival of juvenile *P. maculatus* at the Keppel Islands. When averaged across all years, the conditions associated with spawning activity are only very broadly associated with the Austral summer conditions.

### (c) Aligning seasonal fishing closures with spawning times

**Figure 3.**
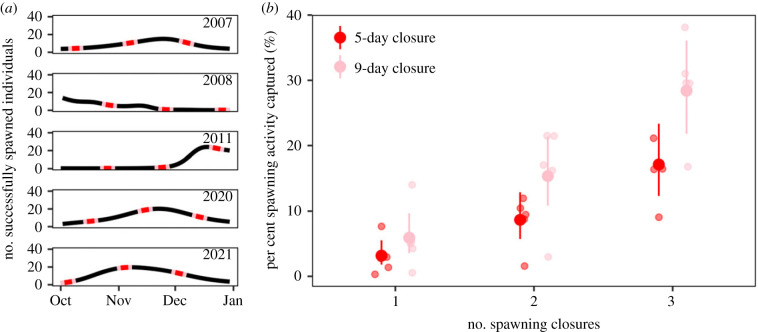
The effectiveness of fishery spawning closures depends on the timing and duration of spawning activity of *P. maculatus* at the Keppel Islands. (*a*) The timing of five-day (red) and nine-day (pink) spawning closures coinciding with the new moon capture different periods of spawning activity from October to December in each year. (*b*) The number and duration of fishery closures between October and December increases the likelihood of capturing a spawning event, thereby increasing the proportion of spawning activity protected by spawning closures in each year and reducing the volatility in their effectiveness. Increasing the number of closures had a greater effect than increasing the duration of seasonal closures.

Using the predicted spawning activity in each year (figure 1), we explored how spawning activity aligned with seasonal closures. In 2007 and 2008, there were 27 days of closures and 10 days in subsequent years—assuming an equal daily probability of spawning, we would expect seasonal closures to capture 29% and 11% of all spawning activity between October and December, respectively. However, temporal spawning patterns were highly variable during these periods (figure 3*a*). In 2007 and 2008, three 9-day spawning closures captured 41.5% and 20.9% of spawning activity, respectively. In 2011, 2020 and 2021, two 5-day spawning closures captured 1.2%, 13.5% and 10.0% of spawning activity, respectively. Spawning closures were not effective at capturing peak spawning activity between October and December, which represents only a fraction of all spawning that occurs year-round.

We investigated whether increasing the number and duration of spawning closures between October and December increases the likelihood of a closure coinciding with capturing a spawning event, thereby increasing the proportion of spawning activity protected by spawning closures in each year. The interaction between the duration and the number of closures was not important, indicating that the effect of duration did not depend on the number of closures, and vice versa. Across years, we found that 9-day closures around the new moon captured 1.9 times more spawning activity than 5-day closures (Tukey's: d.f. = 28, *t* = 3.304, *p* = 0.001, electronic supplementary material, table S5). Although the difference is significant, it is only marginally higher than the expected increase in spawning activity for the additional days of closure (9/5 = 1.8). The number of closures had a larger effect on capturing spawning activity (figure 3*b*; electronic supplementary material, table S5). On average, a single 5-day and 9-day closure in October captured 3.2% (95% CI [1.8–5.5]) and 5.9% (95% CI [3.6–9.7]) of all spawning activity between October and December, respectively. The spawning activity captured by closures increased by 189% with an additional closure in November (Tukey's: *t* = 3.61, *p* < 0.001) and by a further 118% with an additional closure in December (Tukey's: *t* = 3.50, *p* < 0.01). Collectively, longer 9-day closures may not yield an overall net benefit beyond what would be expected for the duration of the closures and multiple closures may be more effective in mitigating the annual and monthly volatility in spawning activity by increasing the likelihood of capturing peaks in successful spawning activity.

## 4. Discussion

High-resolution age and size estimates of juvenile *Plectropomus maculatus* at the Keppel Islands over a 15-year period revealed several unexpected temporal patterns in spawning activity. Despite being a high latitude reef fish population, spawning occurred in all months of the year, with each sampling period showing broad summer and winter cohorts that varied in terms of their exact timing and duration. Unexpectedly, there was no effect of lunar patterns on spawning activity, and we found no clear environmental cue at the onset of spawning peaks. Such lack of seasonality and asynchrony with lunar cycles in the spawning activity of *P. maculatus* suggest the current timing and duration of spawning closures, which are based on spawning observations for *P. leopardus*, are of limited benefit to *P. maculatus* in this region.

It is evident that spawning in *P. maculatus* at the Keppel Islands is occurring in periods of 2–3 months to generate distinct cohorts in the juvenile population than can occur at any time of the year. This was unexpected given that histological assessments of seasonal gonad development suggest most coral reef fishes on the GBR have distinct spawning seasons, usually late spring to early summer [14,22,36]. The spawning patterns found in this study indicate *P. maculatus* contrasts with other reef fishes with restricted summer recruitment periods [1,10,22,56]. They also contrast with courtship and aggregating behaviours, spawning observations, recruitment observations and histological studies of other *Plectropomus* spp. on the GBR, which have been documented around new moon phases in spring and summer [36,57–62]. However, all studies except those of Samoilys [36] and Heupel *et al*. [62] were restricted to spring and summer months (September to February), suggesting winter spawning events may have been missed. While *P. leopardus* and *P. laevis* both exhibit clear seasonal spawning in late spring and early summer on the central and northern GBR [36,62], both studies also indicate some spawning activity during other times of year, which support our findings. While our results were not corroborated with histological assessments of seasonal gonad development, parentage studies have confirmed that the large majority of juvenile *P. maculatus* that successfully recruit to the Keppel Islands originate from local reefs [15,63], and are therefore representative of spawning activity throughout the year.

Year-to-year variation in spawning activity with only a weak association with SST and the time of year indicates other mechanisms not captured in our model may be responsible for the temporal variation in spawning activity of *P. maculatus*. For example, survivorship of pre- and post- settlement larvae [14,29,30] and fluctuations in the abundance of prey abundance throughout the year may lead to differential fitness of juvenile fish [29,30]. Juvenile *P. maculatus* consume small crustaceans and gobies [64,65], which may also fluctuate in abundance. Equally, adult *Plectropomus* spp. are piscivorous [66] and fluctuations in the availability of prey species for breeding female fish may lead to fluctuation in the timing and duration of spawning peaks.

While spawning and recruitment of coral reef fishes often follow a lunar cycle [23,25,28,67], this was clearly not the case for *P. maculatus* at the Keppel Islands. Lunar spawning offers a strong set of environmental cues for synchronizing spawning across a population [14], which has been documented for the closely related *P. leopardus* at Scott Reef on the GBR [36] and extended to *P. maculatus* [38,60]. This assumption probably stems from the observation that *P. maculatus* occasionally appears in lunar spawning aggregations of *P. leopardus* [60]. One hypothesis for the lack of lunar spawning may be due to the increased cost of spawning migrations for lunar cyclic spawning [68,69]. There have been no observations of spawning aggregations of *P. maculatus* at the Keppel Islands or movement between reefs. Individuals are likely to be spawning in small groups that would lead to the more continuous and unsynchronized spawning we observed. Another factor could be geographical variation in the propensity to exhibit lunar spawning patterns. Studies have demonstrated intraspecific variation in lunar spawning patterns in different areas [1,2,10]. Hence, the lack of lunar spawning in *P. maculatus* on the southern GBR may be atypical and does not necessarily apply to populations at lower latitudes.

Temporal volatility in spawning activity has two clear implications for fisheries management. In the case of *P. maculatus* at the Keppel Islands, seasonal spawning closures did not effectively protect spawning activity due to the lack of synchrony with the new moon and year-to-year variation in the timing of peak spawning activity. Seasonal closures are implemented with the primary objective of protecting spawning aggregations vulnerable to overfishing yet are likely too short or too infrequent to effectively capture spawning activity. This suggests that the current temporal closures are too narrow or too few and it is likely that other species managed under the Queensland Line Fishery (Reef) will exhibit similar variation in spawning, which needs to be investigated as a high priority. Secondly, we show that increasing the number of spawning closures may provide greater benefits than increasing their duration in order to increase the likelihood of capturing peaks in spawning activity. While these findings may be useful in guiding revisions for the management plan of *P. maculatus* on the southern GBR, we caution that complex ecological traits such as spawning activity may not be transferable across regions or species, even conspecifics.

It's important to note the Queensland Reef Line Fishery is supplemented by the GBR Marine Park no-take marine reserve network, and directly managed via a range of additional catch and effort controls including minimum size-limits, recreational possession limits, as well as limited entry licencing and total allowable catch quotas for commercial operators. Given the volatility in spawning activity, optimizing such measures to protect a greater proportion of spawning biomass could be of greater benefit to the productivity of fisheries on the GBR than alterations to the timing and duration of seasonal closures. For example, studies of *P. maculatus* at the Keppel Islands have demonstrated how a network of no-take marine reserves [15,63] and minimum size limits [70] can effectively protect spawning biomass, delivering important recruitment contributions to replenish local fished populations. Strengthening these measures could compensate for the ineffectiveness of seasonal closures and uncertainty in life-history traits for a wider range of species.

Ageing of juvenile *P. maculatus* at the Keppel Islands indicates strong variation in the timing of spawning beyond what was previously understood for coral reef fishes. To investigate these patterns further, consideration should be given to the experimental design to accurately assess spawning times of juvenile coral trout, the potential causes of temporal variation in spawning peaks, and whether the patterns observed at the Keppel Islands are consistent throughout the GBR and for other congeners. An important caveat to this study is that our estimates of peak spawning activity are based on juvenile fish that successfully settled and recruited to the island group and may not be representative of all spawning in the region, particularly spawning that leads to unsuccessful recruitment. Although we are confident that our sampling design identified recruitment cohorts for the period that we investigated, there may have been additional cohorts in some years. Ideally, future studies would employ a combination of histological studies of seasonal gonad development and direct observations of spawning behaviour, followed by recruitment surveys, and matched with parentage to provide a direct link between spawning and recruitment. On their own, each approach has offered insights into the behaviour [36], dispersal patterns [63] and reproductive success [70] of groupers. When combined, these methods may offer important new insight into reproductive strategies and the ability to predict peak spawning periods in coral reef fish.

## 5. Conclusion

Our findings highlight several unexpected temporal patterns in the spawning activity of *P. maculatus* at the Keppel Islands. In each year, recruitment originated from several distinct cohorts with no consistent timing or environmental trigger. These patterns suggest a mismatch with management strategies that aim to protect peak spawning activity on the GBR. If species have a bet-hedging reproductive strategy or environmental conditions create volatility in their reproductive success, then fisheries management must adopt strategies that mitigate these uncertainties. In the case of *P. maculatus* at the Keppel Islands, the current two-times 5-day spawning closures were ineffective at protecting successful spawning activity and may need to be extended and/or multiplied to ensure adequate protection of spawning biomass. Our findings demonstrate that there is scope to refine seasonal spawning closures in the Reef Line Fishery to maximize their effectiveness and provide additional complementarity to existing fishery controls and marine park management actions.

## Data Availability

The data are provided in electronic supplementary material and online as live https://github.com/HugoBH/GKI-Spawning-activity.git and static repositories [71].
